# Study on the impact of COVID-19 pandemic on the mental health of Chinese college students: a cross-sectional analysis

**DOI:** 10.3389/fpubh.2024.1340642

**Published:** 2024-04-15

**Authors:** Xiaodong Song, Demin Han, Jiaqi Zhang, Jiajun Fan, Peishan Ning, Yong Peng

**Affiliations:** ^1^School of Traffic and Transportation Engineering, Central South University, Changsha, China; ^2^Joint International Research Laboratory of Key Technology for Rail Traffic Safety, Central South University, Changsha, China; ^3^Department of Epidemiology and Health Statistics, Xiangya School of Public Health, Central South University, Changsha, China

**Keywords:** COVID-19, mental health, college students, social media, mitigation measures

## Abstract

**Background:**

The COVID-19 pandemic has significantly impacted the mental health of college students, prompting the need for universities to implement measures to mitigate these adverse effects. This study aims to assess the mental health status and mitigation measures of college students, identify the primary factors contributing to their mental health challenges, and provide suggestions for educational institutions to reduce negative psychological impacts.

**Methods:**

In February 2023, a questionnaire survey was conducted among 1,445 college students. Statistical analysis was performed on the survey results, and multiple regression models were used to identify significant influencing factors and optimize the model.

**Results:**

The study revealed correlations between factors affecting mental health during the pandemic, with interactions observed among some factors. Significant differences in mental health status were found among different groups of college students based on their information-sharing habits through apps and engagement in thesis research. Multiple regression analysis indicated that conducting academic research related to COVID-19 significantly increased the psychological stress of college students during the pandemic (*p* = 0.043). Among all mitigation measures, playing games demonstrated significant effectiveness in model analysis (*p* = 0.047). The optimization of the model showed that the multiple regression model considering the interaction of factors was more effective.

**Conclusion:**

Our research identifies crucial factors influencing the mental health of college students and investigates the mental health status of various student groups. We recommend that educational institutions adopt proactive strategies and a multifaceted approach to support the mental health of college students and address potential issues that may arise.

## 1 Introduction

The COVID-19 pandemic, first identified in Wuhan, China, at the end of 2019, subsequently spread globally, leading to significant morbidity and mortality. Recognized for its high transmissibility and various health impacts, the World Health Organization (WHO) officially declared it a global pandemic on March 11, 2020. In response, governments and public health institutions worldwide implemented various measures to contain the virus and safeguard public health.

During this period, the disease not only posed a great threat to physical health but also imposed a considerable psychological burden. As noted by Hossain et al. (1) in their review, the COVID-19 pandemic led to the emergence of mental health issues such as depression, anxiety, stress, anger, emotional disturbances, and post-traumatic stress, reflecting factors related to mental health problems like age, gender, residence, and coping strategies. This underscores the necessity of implementing mental health interventions for the population. As a specific group, college students’ mental health issues are considered a crucial public health concern. Stallman’s (2) research mentioned that the prevalence of mental health problems among college students is significantly higher than in the general population, with major contributing factors including employment status and economic pressures. During the pandemic, changes in the employment environment and social status, along with online learning, isolation, and financial difficulties, may exacerbate existing mental health issues among college students. For instance, Ghazawy et al. (3) ER’s survey on Egyptian college students’ mental health found that 70.4% of students suffered from depression, 53.6% from anxiety, and 47.8% experienced high levels of stress. A study on perceived stress in Turkey indicated that more than half of the students met the diagnostic criteria for anxiety (52%) and depression (63%) (4).

Several studies have analyzed factors related to college students’ mental health problems. Sujarwoto et al. (5) and Nguyen-Feng et al. (6) found that increased use of social media by college students heightened their psychological vulnerability, leading to depressive states. Ochnik et al. (7) discussed the differences in mental health issues among college students based on gender, while Sunna’s research (8) showed that female students faced more severe mental health problems than male students, possibly due to higher levels of perceived stress (9). During the pandemic, various sources contributed to the widespread mental health issues among college students. Son et al.’s (10) interviews revealed that the main stressors causing symptoms like depression and anxiety included concerns about personal and family health, sleep disorders, and reduced social interactions. Chen and Lucock’s (11) study in Northern England identified decreased levels of exercise and lifestyle changes as the main causes of student depression. Fila-Witecka et al. (12) also emphasized the impact of lifestyle changes on college students’ mental health.

In China, the spread of COVID-19 can be divided into two phases: the first phase, from the outbreak at the end of 2019 to the end of 2022, where large-scale infections did not occur domestically due to relatively stricter management measures compared to abroad; and the second phase, from the change in pandemic policies in December 2022 to the present, where the strictness of management measures has been reduced, leading to the widespread transmission of COVID-19 within the country and an increase in the complexity of mental health issues. In the first phase, Sun et al. (13) and Yu’s et al. (14) cross-sectional survey found that lack of social support and stigmatization were the main sources of mental health problems, while Ma et al. (15) used the Event Impact Scale-6 and the Patient Health Questionnaire-9 to identify concerns about family and friends’ infections, media coverage, and low social support as the main influencing factors. Jiang (16) posited that insufficient understanding of COVID-19 and the perception of the virus’s risks impacted their mental health. In the second phase, Song et al. (17) explored the relationship between academic performance and mental health status after lifting restrictions, indicating that college students’ mental health continued to be affected by COVID-19 during this phase. Deng et al. (18) examined the relationship between internet addiction and mental health issues among college students after the lifting of restrictions, highlighting the need for establishing corresponding social intervention measures.

It is evident that the mental health issues of Chinese college students during the first phase were influenced by various factors, including the level of social support, concerns for others, understanding of COVID-19, and academic performance. However, in the second phase, after the lifting of restrictions, existing research has the following shortcomings in facing the more complex mental health issues of college students:

(1) The exploration of factors influencing mental health is relatively singular;

(2) While the importance of mitigation measures and social support is highlighted, there is a lack of focus on what specific mitigation measures should be taken by college students facing mental health issues.

To the best of the authors’ knowledge, there has been a lack of research focusing on the role of mitigation measures and specific recommendations from schools in the context of mental health issues among Chinese college students. Therefore, this study employs a cross-sectional survey method to collect online questionnaires, aiming to identify the most critical factors influencing college students’ mental health, understand the mitigation measures they adopt, determine the most effective ways of alleviating these issues, and provide concrete suggestions for schools to improve the mental well-being of their students.

## 2 Methods

Our research study, aimed at evaluating the mental health status of a cohort of Chinese college students, was conducted using “WenJuanxing,” a leading questionnaire software in China. The investigation was carried out over a month-long period through meticulously designed questionnaires, the questionnaire was completed by collecting online from students of Central South University (CSU). For the acquired data, we performed correlation, difference, and multiple linear regression analysis using SPSS 26.0 (19).

### 2.1 Questionnaire design

#### 2.1.1 Personal information

In our research, we gathered personal information such as gender (male and female), academic year, and place of origin. We divided the academic year into six categories: freshmen, sophomores, juniors, seniors, master’s students, and doctoral students. The origin of the students was classified based on the official ranking of Chinese cities, providing insights into potential regional differences in mental health status (20, 21).

#### 2.1.2 Attention on COVID-19

We developed a comprehensive questionnaire to ascertain participants’ attention to the epidemic, including their sources of information (22), duration and breadth of attention, and concern about its societal impact. Popular information sources like news reports, public notifications, and app shares were included. The duration of attention was assessed with a single-choice question, ranging from less than 3 h to over 24 h per week. The focus of attention was divided into various aspects, such as the virus’s infectiousness, fatality rate, long-term effects, and potential for mutation (23). Social impact concerns covered medical care, employment conditions, etc. Apart from duration, all questions were multiple-choice with an “other” option for unspecified concerns.

#### 2.1.3 Mental health status

Participants’ mental health during the epidemic was assessed using a custom 20-question scale based on the Symptom Checklist-90 (22) (SCL-90) depression scale. Responses ranged from “very good agreement” to “very bad agreement,” scored from 1 to 5, with higher scores indicating better mental health status. The SCL-90 has been effective in assessing depression and anxiety symptoms in Chinese cohorts.

#### 2.1.4 Measures and recommendations

We surveyed participants about their methods of mitigating adverse (24) mental health conditions, assessing both employed methods and their recommendations, such as talking, exercising, sleeping, eating, etc. Participants rated these approaches on a Likert scale from 1 (completely ineffective) to 5 (very effective). We also solicited suggestions for ways schools could aid in alleviating psychological conditions (25).

### 2.2 Investigation procedure

This study was conducted during an epidemic outbreak when most college students in China were in home quarantine. Data was collected via an online survey completed by 1,444 students from a specific college at a certain university, conducted over a 10-day period. With assistance from college counselors, we distributed the survey via web links. Of the 1,445 responses, 1,309 were valid, resulting in a 91% effective response rate. The Ethics Committee of Central South University approved this study, conducted via an anonymous online survey.

### 2.3 Statistical analysis

We statistically analyzed the data procured from the questionnaire. Preliminary evaluation of data normality revealed that both mental health status scores and mental health levels displayed a non-normal distribution.

Firstly, we conducted a correlation analysis (26) among variables comprising sociodemographic characteristics, levels of attention, and measures to alleviate adverse psychological conditions. This exercise yielded a network of variable correlations.

Secondly, we conducted descriptive statistics to determine the proportion of various groups (27) and compute the mean mental health scores per group. We also utilized a non-parametric test to investigate the disparities in mental health scores among different groups. Factors under consideration included gender, age, place of origin, and duration of concern. Moreover, we evaluated differences based on the choice of various sources and content of virus-related concern, perceived social influence, and chosen methods of mitigation.

Lastly, we sought to investigate the influence of each variable on mental health status scores via a three-step multiple linear regression analysis (28, 29). The initial step involved the analysis of all potential influencing variables and identification of significantly impacting factors. During the construction of the multiple linear regression model (Model 1), we incorporated four interaction variables: interaction among channels of concern, content of concern about the virus and its social impact, measures to mitigate poor mental health conditions, and demographic variables.

In the second step, we retained significant variables from the first step into Model 2. In the final step, to verify the interaction term’s impact on the model and its significance on mental health status scores, we incorporated the previously mentioned four interaction variables into Model 2 for analysis.

The linear regression analysis results included the unstandardized coefficient *B* values, standard deviation (SD), 95% confidence interval (CI), standardized coefficient beta, and significance result p of the variables. ANOVA was employed for model comparison, aiding in the determination of the interaction term effect. All regression analyses adhered to the assumptions concerning the distribution of the variables (30).

## 3 Results

### 3.1 Correlation statistics

**Figure 1:**
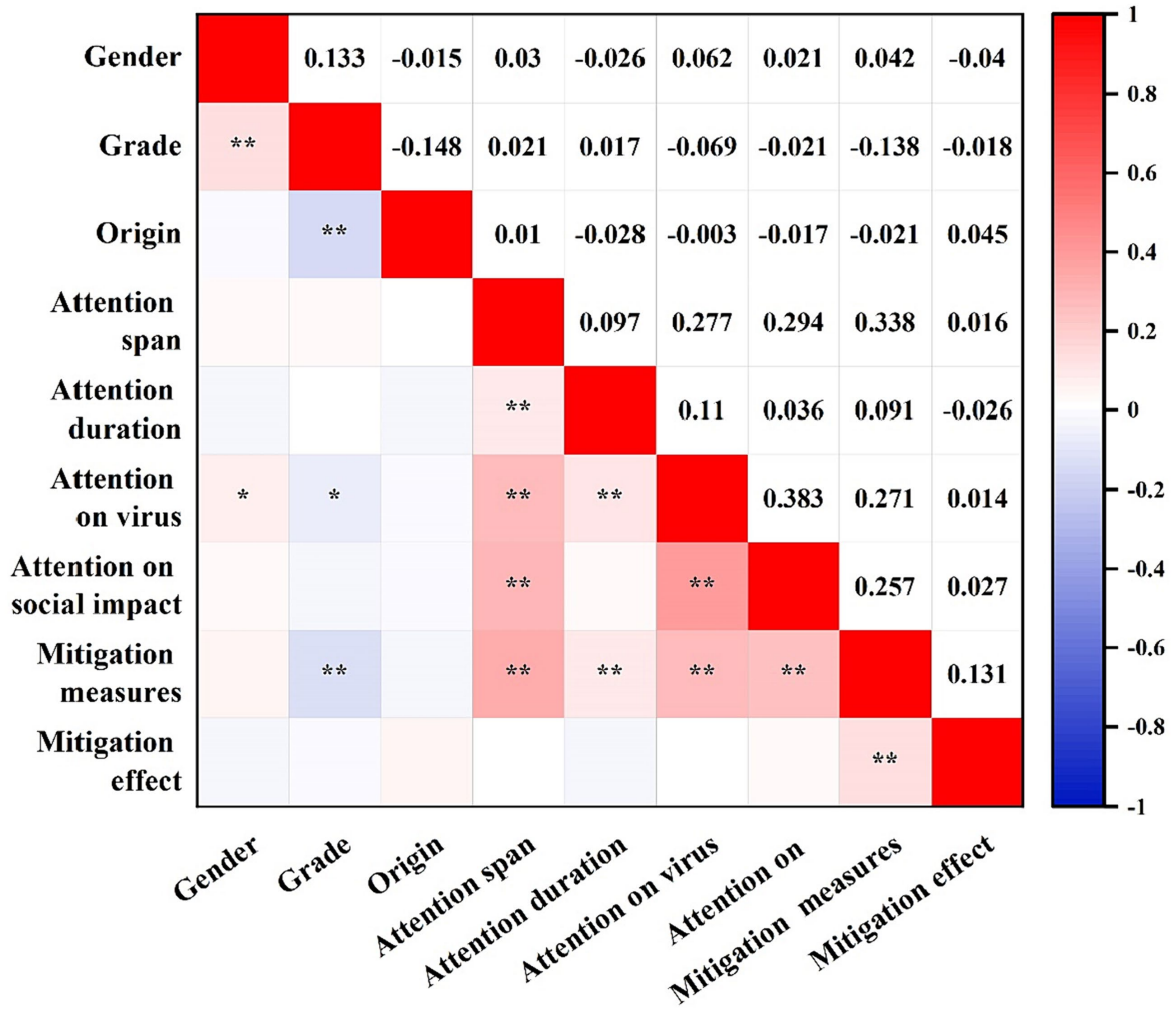
Diagram of Pearson’s correlation coefficient among variables.

Figure 1 displays the results of our correlation analysis between variables, with significant correlations (*p* < 0.05) between several variables. The duration that participants dedicated to infectious disease information was significantly associated with the breadth of concern (*p* < 0.01). Additionally, the nature of participants’ concern about the virus showed a significant correlation with age, gender, breadth and length of concern (*p* < 0.05, *p* < 0.01). The perceived social impact of the virus correlated strongly with both the breadth of concern and the specific content of concern about the virus (*p* < 0.01). Furthermore, the strategies employed by participants to mitigate adverse mental health conditions showed a strong correlation with their grade and all concern variables (*p* < 0.01). Lastly, the efficacy of these mitigation measures demonstrated a significant correlation with the mitigation methods employed (*p* < 0.01).

### 3.2 Descriptive and differential statistics

Our analysis was based on 1,309 validated questionnaires. The mean of the mental health scores of all participants was 61.980. Table 1 presents the distribution of participants according to gender, academic level, and place of origin, along with the corresponding mean mental health scores for each group. The table also reveals no significant differences among various sociodemographic groups.

**Table 1:** Information of participants with different average mental health scores.

Sociodemographic variables	*N*(%)	Average score	*p*-value
Amount, *n*(%)	1,309 (100.0)	61.983	
Gender, *n*(%)			0.320
Male	648 (49.5)	62.093	
Female	661 (50.5)	61.876	
Grade, *n*(%)			0.724
Freshman	441 (33.7)	61.730	
Sophomore	418 (31.9)	61.813	
Junior	80 (6.1)	62.750	
Senior	81 (6.2)	62.728	
Master’s student	258 (19.7)	62.213	
Ph.D. student	31 (2.4)	62.032	
Origin, *n*(%)			0.445
First-tier city	20 (1.5)	61.500	
New-tier city	331 (25.3)	62.130	
Second-tier city	166 (12.7)	61.163	
Third-tier city	323 (24.7)	62.474	
Fourth-tier city	298 (22.8)	61.685	
Fifth-tier city	171 (13.1)	62.146	

Table 2 presents the distribution and mean values of participants’ sources, durations, epidemic-related content, and social implications of their concerns. News reports were the predominant source of concern for the majority of participants (92.1%), while a smaller group (12.5%) turned to academic research. Many participants (87.2%) spent less than 3 h weekly focusing on epidemic concerns. Regarding concerns specific to the epidemic, post-infection sequelae and infectivity were major issues for 89.4 and 80.4% of participants, respectively. In terms of the epidemic’s social implications, healthcare concerns were foremost (86.6%), followed by industry changes (74.3%) and employment pressures (70.4%). Statistically significant differences were found in mental health scores among various subgroups. For example, those who followed news through app-sharing (*p* = 0.03) and academic research (*p* = 0.002) showed notable differences. Significant disparities were also observed in concerns related to the epidemic’s mortality rate (*p* = 0.025), and social implications like industry changes (*p* = 0.001) and employment pressures (*p* = 0.007).

**Table 2:** Attention of participants with different average mental health scores.

Attention level	*N*(%)	Average score	*p*-value
Attention source, *n*(%)			
News report			0.061
Yes	1,205 (92.1)	61.868	
No	104 (7.9)	63.317	
Public account			0.103
Yes	1,023 (78.2)	61.836	
No	286 (21.8)	62.51	
APP sharing			0.030^*^
Yes	653 (49.9)	61.605	
No	656 (50.1)	62.360	
Discussion with others			0.320
Yes	962 (73.5)	61.918	
No	347 (26.5)	62.164	
Thesis research			0.002^**^
Yes	163 (12.5)	60.724	
No	1,146 (87.5)	62.162	
Others			0.322
Yes	5 (0.4)	60.000	
No	1,304 (99.6)	61.99	
Attention duration, *n*(%)			0.090
<3 h per week	1,141 (87.2)	62.089	
3–12 h per week	146 (11.2)	61.267	
12–24 h per week	13 (1.0)	59.385	
>24 h per week	9 (0.7)	63.889	
Attention on virus, *n*(%)			
Infectivity			0.084
Yes	1,053 (80.4)	61.816	
No	256 (19.6)	62.272	
Lethality			0.025^*^
Yes	822 (62.8)	61.680	
No	487 (37.2)	62.495	
Sequelae			0.649
Yes	1,170 (89.4)	61.967	
No	139 (10.6)	62.122	
Variation			0.375
Yes	797 (60.9)	61.828	
No	512 (39.1)	62.225	
Others			0.619
Yes	25 (1.9)	64.280	
No	1,284 (98.1)	61.938	
Attention on social impact, *n*(%)			
Medical security			0.263
Yes	1,134 (86.6)	61.915	
No	175 (13.4)	62.423	
Industry change			0.001^**^
Yes	973 (74.3)	61.657	
No	336 (25.7)	62.929	
Employment pressure			0.007^**^
Yes	921 (70.4)	61.711	
No	388 (29.6)	62.629	
Others			0.136
Yes	28 (2.1)	63.750	
No	1,281 (97.9)	61.945	

^*^p < 0.05, ^**^p < 0.01.

Table 3 illustrates the various mitigation strategies adopted by participants, the effectiveness of these strategies, and their mean values. The most commonly employed strategy was sleeping (79.2%), and a majority of participants (94.3%) reported some level of emotional mitigation from their chosen methods. The results revealed significant variations in mental health scores among groups choosing to talking and sharing (*p* = 0.009), eat (*p* = 0.001), study (*p* = 0.024), recreate outdoors (*p* = 0.023), listen to music (*p* = 0.049), and watch movies (*p* = 0.028) as their mitigation strategies. Furthermore, the perceived effectiveness of these strategies showed highly significant intergroup variability (*p* = 0.005).

**Table 3:** Mitigation of participants with different average mental health scores.

Mitigation measures and effects	*N*(%)	Average score	*P*-value
Measures, *n*(%)			
Talking and sharing			0.009^**^
Yes	813 (62.1)	61.649	
No	496 (37.8)	62.530	
Exercising			0.101
Yes	785 (60.0)	61.731	
No	524 (40.0)	62.361	
Sleeping			0.712
Yes	1,037 (79.2)	61.923	
No	272 (20.7)	62.210	
Eating			0.001^**^
Yes	581 (44.4)	61.466	
No	728 (55.6)	62.397	
Study			0.024^*^
Yes	404 (30.9)	61.401	
No	905 (69.1)	62.243	
Retaliatory consumption			0.186
Yes	163 (12.5)	61.448	
No	1,146 (87.5)	62.059	
Travel			0.169
Yes	357 (27.3)	61.664	
No	952 (72.7)	62.103	
Outing entertainment			0.023^*^
Yes	566 (43.2)	61.638	
No	743 (56.7)	62.246	
Listening to music			0.049^*^
Yes	810 (61.9)	61.763	
No	499 (38.1)	62.341	
Watching movies			0.028^*^
Yes	610 (46.6)	61.613	
No	699 (53.3)	62.306	
Playing games			0.449
Yes	664 (50.7)	62.181	
No	645 (49.3)	61.780	
Other Mitigation measures			0.156
Yes	10 (0.8)	59.700	
No	1,299 (99.2)	62.001	
Effects, *n*(%)			0.005^**^
Very ineffective	18 (1.4)	54.389	
Relatively ineffective	56 (4.3)	61.071	
Ordinary	328 (25.1)	62.857	
Relatively effective	610 (46.6)	62.116	
Very effective	297 (22.7)	61.377	

^*^p < 0.05, ^**^p < 0.01.

**Figure 2:**
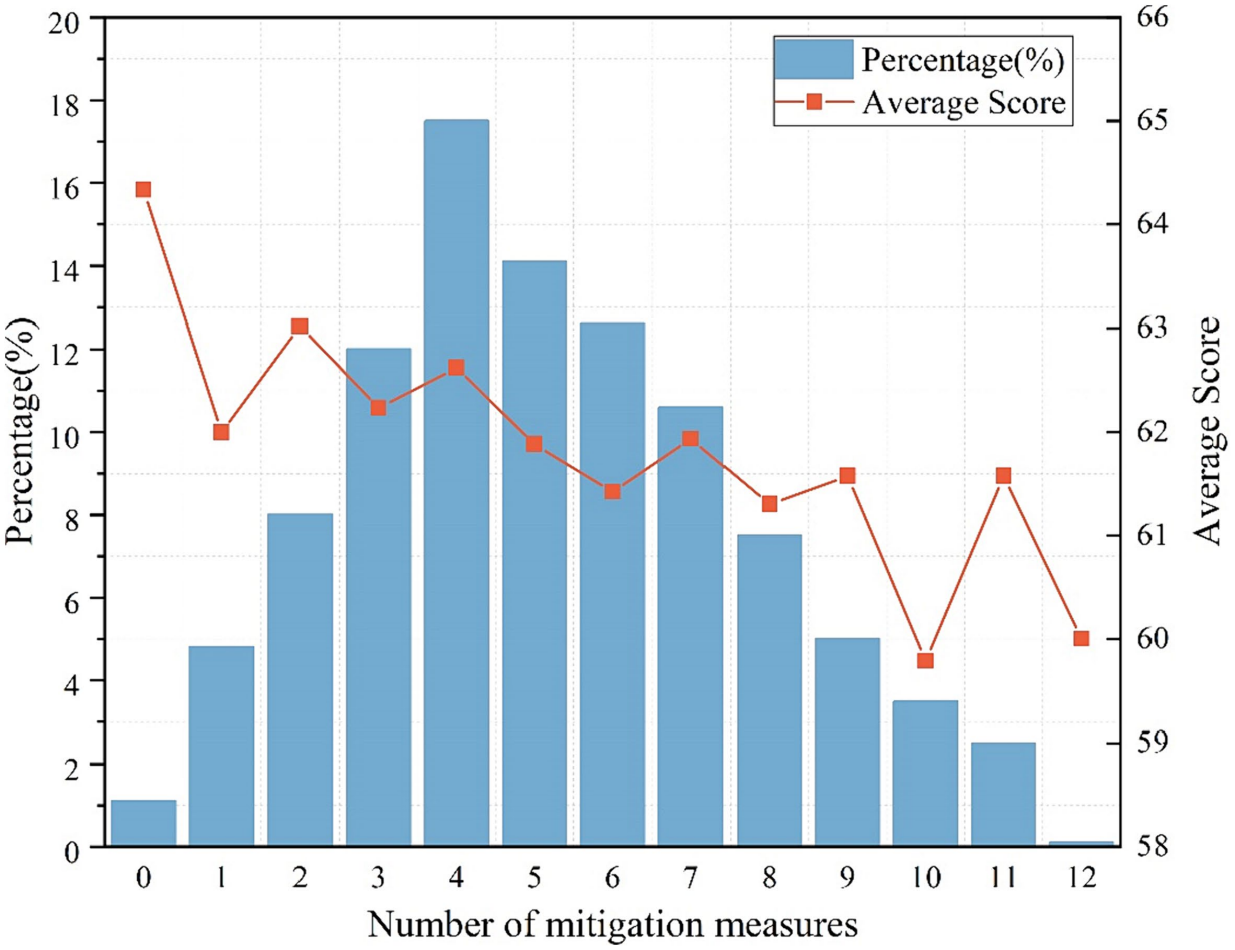
Proportion of participants with different numbers of mitigation measures and mental health scores.

Our research uncovered an intriguing observation: participants opting for various mitigations tended to have poorer mental health. This trend might be attributed to the likelihood that individuals already experiencing mental health issues were more inclined to choose these mitigations. To substantiate this hypothesis, we analyzed the correlation between the number of mitigations selected and participants’ average mental health scores, as depicted in Figure 2.

We observed that participants who did not adopt any mitigation measures reported the highest average mental health scores. Interestingly, there was a discernible pattern: as the number of mitigations chosen by a participant increased, their average mental health scores correspondingly declined. Utilizing a non-parametric test, we established that the differences across these groups were statistically significant (*p* = 0.042), thus confirming our initial hypothesis.

### 3.3 Multiple linear regression analysis

Table 4 presents the analysis results of the multiple linear regression model (Model 1), which encompasses all potential variables and interaction terms. As determined by the *p*-values, four variables significantly impacted mental health scores: the focus on thesis research related to infectious diseases (*p* = 0.043), the attention given to additional aspects of the infectious disease virus (*p* = 0.047), the consideration of other social impacts resulting from the infectious disease (*p* = 0.019), and the utilization of gaming as a mitigation strategy for poor mental health (*p* = 0.047).

**Table 4:** Predictors of mental health status of Model 1.

	*B*	95%Cl	*SD*	Beta	*p-*value		*B*	95%Cl	*SD*	Beta	*p-*value
Characteristics											
Gender	0.325	[−0.658,1.308]	0.501	0.026	0.517	Talking and Sharing	−0.693	[−1.447,0.062]	0.385	−0.054	0.072
Grade	0.302	[−0.162,0.766]	0.237	0.077	0.202	Exercising	−0.051	[−0.807,0.705]	0.386	−0.004	0.895
Origin	0.093	[−0.266,0.452]	0.183	0.021	0.612	Sleeping	0.311	[−0.588,1.21]	0.458	0.02	0.497
Attention by news reports	−1.08	[−2.356,0.195]	0.65	−0.047	0.097	Eating	−0.665	[−1.398,0.067]	0.373	−0.053	0.075
Attention by public account	−0.267	[−1.135,0.600]	0.442	−0.018	0.546	Study	−0.46	[−1.266,0.345]	0.41	−0.034	0.262
Attention by APP sharing	−0.258	[−0.995,0.479]	0.376	−0.021	0.493	Retaliatory consumption	−0.423	[−1.593,0.747]	0.596	−0.022	0.479
Attention by discussion	0.283	[−0.525,1.092]	0.412	0.02	0.492	Travel	0.274	[−0.594,1.143]	0.443	0.02	0.536
Attention by thesis research	−1.161	[−2.287,-0.034]	0.574	−0.061	0.043^*^	Outing entertainment	−0.373	[−1.173,0.427]	0.408	−0.03	0.361
Attention by other channels	−3.219	[−12.046,5.067]	4.499	−0.032	0.474	Listening to music	−0.213	[−1.034,0.608]	0.418	−0.017	0.611
Attention duration	−0.177	[−0.974,0.621]	0.406	−0.012	0.664	Watching movies	−0.374	[−1.171,0.423]	0.406	−0.03	0.357
Attention on infectivity	−0.356	[−1.261,0.549]	0.461	−0.023	0.440	Playing games	0.807	[0.011,1.603]	0.406	0.065	0.047^*^
Attention on lethality	−0.275	[−1.031,0.481]	0.385	−0.021	0.476	Other Mitigation measures	−2.541	[−6.559,1.476]	2.048	−0.035	0.215
Attention on sequelae	0.669	[−0.502,1.840]	0.597	0.033	0.262	Mitigation effect	0.151	[−0.249,0.552]	0.204	0.021	0.459
Attention on variation	0.227	[−0.535,0.988]	0.388	0.018	0.559						
Attention on other factors	2.752	[0.034,5.470]	1.386	0.06	0.047^*^	Interaction items					
Attention on medical security	0.08	[−1.001,1.162]	0.551	0.004	0.884	Interaction 1(span)	3.76	[−9.189,16.708]	6.600	0.029	0.569
Attention on industry change	−0.717	[−1.570.0.136]	0.435	−0.05	0.099	Interaction 2(impact)	−9.298	[−19.305,0.708]	5.101	−0.071	0.069
Attention on employment pressure	−0.285	[−1.116,0.546]	0.424	−0.021	0.502	Interaction 3(measures)	0.601	[−0.035,1.236]	0.324	0.06	0.064
Attention on other social impacts	3.103	[0.513,5.694]	1.32	0.072	0.019^*^	Interaction 4(demography)	−0.028	[−0.099,0.042]	0.036	−0.056	0.425

^*^p < 0.05.

Table 5 illustrates the multiple linear regression results for Model 2, which was derived from significant factors, and Model 3, which incorporated the interaction terms. In Model 2, the focus on dissertation research related to infectious diseases emerged as a significant determinant of mental health status (*p* = 0.002). The addition of interaction terms in Model 3 expanded the number of variables with significant effects. Interestingly, Model 3 (*R*^2^ = 0.017, *F* = 4.026) exhibited superior performance over Model 2 (*R*^2^ = 0.012, *F* = 2.812), underscoring the pivotal role of interaction terms in enhancing the multiple linear regression model. However, these interaction terms did not yield a significant influence on the cumulative mental health score.

**Table 5:** Predictors of mental health status of Model 2 and Model 3.

	*B*	95%Cl	*SD*	Beta	*p-*value		*B*	95%Cl	*SD*	Beta	*p-*value
Model 2						Model 3					
Attention by thesis research	−1.662	[−2.695,−0.629]	0.526	−0.088	0.002^**^	Attention by thesis research	−1.605	[−2.660,-0.550]	0.538	−0.085	0.003^**^
Attention on other factors	2.387	[−0.124,4.898]	1.28	0.052	0.062	Attention on other factors	3.344	[0.711,5.978]	1.342	0.073	0.013^*^
Attention on other social impacts	1.704	[−0.669,4.077]	1.21	0.039	0.159	Attention on other social impacts	2.554	[0.052,5.056]	1.275	0.059	0.045^*^
Playing games	0.462	[−0.215,1.14]	0.345	0.037	0.181	Playing games	0.468	[−0.236,1.172]	0.359	0.037	0.192
						Interaction 1(span)	0.744	[−8.847,10.335]	4.889	0.006	0.879
						Interaction 2(impact)	−10.196	[−20.098,−0.293]	5.048	−0.078	0.044
						Interaction 3(measures)	0.186	[−0.374,0.745]	0.285	0.019	0.515
						Interaction 4(demography)	0.008	[−0.020,0.036]	0.014	0.016	0.583

^*^p < 0.05, ^**^p < 0.01.

## 4 Discussion

To our knowledge, this cross-sectional study aligns with past research conducted both in China and globally, which explored the mental health status of college students during infectious disease pandemics. Our research advances previous work by specifically examining mental health status within the context of such pandemics, while conducting comprehensive analyses (correlation, differentiation, and significance) on potential contributing factors to poor mental health. These analyses led to the identification of significant determinants contributing to mental health status and allowed us to explore the influence of variable interactions on the results.

In terms of variable correlations (26), we observed differing relationships (positive versus negative correlations) between different pairs of variables. Our analysis indicated a distinct correlation between participant concerns during the epidemic period. For instance, the length and breadth of concern correlated, suggesting that the number of channels used by participants to follow infectious disease information influenced the duration of concern; more channels were associated with longer periods of attention. As the breadth of attention increased, so did the focus on the virus itself and its societal impact. This is likely due to the fact that broader attention enables the absorption of more information.

The correlation analysis showed that: participants’ concern for the virus itself was positively correlated with gender, and Kecojevic et al. (9) study showed that females showed more concern for COVID-19 during the pandemic period, confirming the inference that higher levels of concern also led to poorer mental health, which is consistent with Xiong’s findings (31). And the number of mitigations was negatively correlated with grade level. It may be because students in higher grades need to devote more time to their studies or employment, resulting in a more homogenous choice of mitigation, as Ravindra Kumar mentioned in his study that academic stress was significantly higher in higher grades than in lower grades (32). Interestingly, we found that participants’ concern about COVID-19 was positively correlated with the number of mitigations taken, which may be due to the fact that the number of mitigations taken by the participants increased with the level of concern. This result can be explained by the fact that as the level of attention increases, participants’ knowledge of relevant information increases, leading to an unhealthy mental state and measures must be taken to alleviate it (33).

Our analysis notably highlighted inter-group differences, focusing on areas where significant disparities were found. The results indicated that participants who accessed information through app sharing had significantly lower mean mental health scores than those who did not use this measure. This finding aligns with Gao et al. (34) and Freiling’s et al. (35) study and further supports the impact of social media use on mental health, as greater exposure to relevant information was associated with poorer mental health status. Additionally, there were highly significant differences in mental health between participants who engaged in academic research and those who did not. Consistent with Luo et al.’s (36) study, individuals involved in COVID-19 research (e.g., healthcare workers) exhibited worse psychological states compared to the general population. For university students, academic research led to a deeper understanding of COVID-19, resulting in increased anxiety and fear.

Regarding concerns about different aspects of the virus, participants who were more worried about the lethality of the virus demonstrated poorer mental health (37). This concern may have intensified their psychiatric issues, leading to heightened fear and worry. In terms of social impact, there was a significant difference between participants who were concerned about changes in the industry and employment pressures and those who were not. Given that the participants in this study were college students, who already had some concerns about employment (10), the pandemic further exacerbated their anxiety.

The outcomes from the multiple linear regression underscored the significance of different variables. The incorporation of interaction terms in the model recognized that potential interactions between the variables, as established in the questionnaire, could be influential. These interactions were noteworthy in the regression analysis, thereby necessitating their inclusion (13).

In Model 1, four pivotal variables had a substantial effect on mental health scores. The participants’ engagement in thesis research was especially impactful, given its academic credibility, which offered a comprehensive understanding of the virus, thereby influencing mental health. Similarly, the participants’ concerns about the virus and its societal implications significantly affected mental health, with “other” content, such as “whether school resumed normally,” also having a strong influence. Interestingly, the act of playing games to alleviate psychological distress showed a notable positive effect on mental health scores. The results of Model 2 further emphasized the substantial effect of thesis research on mental health scores. Among the four significant factors in this model, the impact of thesis research was the most profound, altering the participants’ subjective perceptions through increased understanding.

In order to evaluate the impact of interaction terms, Model 3 was developed by adding four interaction terms to Model 2. Model 3 outperformed Model 2, as the inclusion of interaction terms improved the model’s predictive accuracy for mental health score outcomes. Therefore, when exploring the correlation between each influencing factor and mental health problems, attention should also be paid to the interaction between each influencing factor.

Besides the aforementioned analysis, the survey data incorporated participants’ recommendations on actions universities could undertake. Over half of the respondents advocated for more extracurricular activities and proactive student mental health check-ins by the universities. These findings indicated a positive student response toward such initiatives, coupled with a desire for universities to take a more active role in monitoring their mental health and providing support. This gives crucial insight into the steps universities should consider in the face of similar epidemic situations in the future (3).

## 5 Conclusion

In summary, this study contributes to the exploration of mental health issues among Chinese college students during the pandemic. Firstly, we examined the impact of multiple factors on mental health. The significant effect of thesis research on mental health highlights the relationship between awareness of the virus and mental well-being. Secondly, we investigated the various mitigation measures college students employ to address psychological challenges, finding that gaming is the most effective in alleviating mental health concerns. We also gathered students’ suggestions for the school, providing insights for the formulation of corresponding auxiliary intervention measures. These findings not only enhance our understanding of the complex issue of college students’ mental health but also have practical significance in guiding schools to adopt strategies to address potential problems.

However, our study has limitations. The use of the “Wen Juanxing” methodology, prevalent in China, and the focus on Central South University students may limit the generalizability of our findings. Additionally, participants’ responses might be influenced by their educational backgrounds or perceptions of mental health, potentially leading to an overly positive self-assessment of their mental state, which may not accurately represent the broader psychological condition of college students.

Based on our research results, we advocate for the adoption of proactive strategies, not only recognizing the role of gaming in addressing college students’ mental health problems but also actively promoting virus-related knowledge to reduce students’ panic to a certain extent. Moreover, schools should conduct more social activities and psychological assessments to address potential issues that may arise.

## Data availability statement

The raw data supporting the conclusions of this article will be made available by the authors, without undue reservation.

## Ethics statement

The Ethics Committee of Central South University approved this study (No. 202107008). Written informed consent from the participants was not required to participate in this study in accordance with the national legislation and the institutional requirements.

## Author contributions

XS: Conceptualization, Data curation, Formal analysis, Investigation, Methodology, Software, Writing – original draft. DH: Conceptualization, Data curation, Methodology, Writing – original draft. JZ: Data curation, Writing – original draft. JF: Data curation, Writing – original draft. PN: Methodology, Supervision, Writing – original draft. YP: Funding acquisition, Methodology, Supervision, Writing – original draft.
